# Hydroxyapatite and demineralized calf fetal growth plate effects on bone healing in rabbit model

**DOI:** 10.1007/s10195-014-0323-x

**Published:** 2014-10-12

**Authors:** Amin Bigham-Sadegh, Iraj Karimi, Mohamad Shadkhast, Mohamad-Hosein Mahdavi

**Affiliations:** 1 Department of Veterinary Surgery and Radiology, Faculty of Veterinary Medicine, School of Veterinary Medicine, Shahrekord University, Shahrekord, Iran; 2 Veterinary Pathology, School of Veterinary Medicine, Shahrekord University, Shahrekord, Iran; 3 Veterinary Histology, School of Veterinary Medicine, Shahrekord University, Shahrekord, Iran; 4 School of Veterinary Medicine, Shahrekord University, Shahrekord, Iran

**Keywords:** Hydroxyapatite, Demineralized calf fetal growth plate, Bone healing, Rabbit

## Abstract

**Background:**

Synthetic hydroxyapatite (HA), beta-tricalcium phosphate (β-TCP) and their composite are promising biomaterials, specifically in the orthopedic and dental fields, as their chemical composition is similar to that of bone. Due to the need for safer bone graft applications, these bone graft substitutes are gradually gaining increased acceptability. To stimulate the process of bone healing, several methods have been used previously, including ultrasound, electrical stimulation, exposure to electromagnetic fields, bone grafts, interporous hydroxyapatite (as a bone graft substitute) and bone growth factors. The following study was designed to evaluate the effects of the concurrent usage of hydroxyapatite with demineralized calf fetal growth plate (DCFGP) on the bone healing process.

**Materials and methods:**

Fifteen female New Zealand white rabbits were used in this study. A mid-radius bone defect was created and in the first group (*n* = 5) was filled with hydroxyapatite, in the second group (*n* = 5) with hydroxyapatite and DCFGP, and finally in the third group (*n* = 5) with DCFGP alone. Radiological and histopathological evaluations were performed blindly and the results scored and analyzed statistically.

**Results:**

There was a significant difference for bone formation and remodeling at the 8th post-operative week radiographic assessment (*P*< 0.05), when the hydroxyapatite–DCFGP group was superior to other groups. On the contrary, macroscopical and histopathological evaluation did not revealed significant differences between the three groups

**Conclusion:**

Given the contrasting results of the radiographic assessment and the macro-/microscopic analysis of the healing response, further studies are needed before considering DCFGP-HA as a feasible alternative to HA alone, especially considering the potential hazards and costs of animal-derived biomaterials.

**Level of evidence:**

Not applicable.

## Introduction

There is a continuing search for bone substitutes to avoid or minimize the need for autogenous bone grafts. The use of bone grafts in the management of nonunion cases is well accepted. These grafts act as scaffolds which provide the necessary biomechanical strength that is required to withstand the compressive forces involved during motion. They also promote the ingrowth of cells and other biological products, which eventually leads to the replacement of these grafts by bioactive tissues [1]. Autografts are most widely used by surgeons. These grafts contain viable cells such as bone marrow osteoprogenitor cells, a collagenous matrix and noncollagenous extracellular growth and differentiating factors. Consequently, the autograft is the pre-eminent therapy for bone repair, because it is capable of osteogenesis, osteoinduction and osteoconduction. However, a number of disadvantages such as morbidity in the donor site, the need for general anesthesia or sedation, and the occasional need for more than one surgical field have previously been described in the application of autografts. In addition, graft survival is unpredictable, its resorption cannot be foretold and its availability is limited [2, 3]. It is for these reasons that in recent years several biocompatible materials have emerged as substitutes for autologous bone. Biocompatible materials can be classified into two major organic and synthetic groups. Biological biomaterials can be allogeneic or homologous (human cortical bone and demineralized bone matrix or demineralized freeze-dried), heterologous or xenogeneic (organic bovine, porcine, caprine or coral-derived hydroxyapatite) and replicating (bone morphogenetic proteins; BMPs) [4]. Of the synthetic biomaterial applications of artificial or synthetic hydroxyapatite, bioglasses and bioceramics are more common in orthopedic surgery [5].

Recently, BMPs have been used in clinical trials to enhance bone healing properties [6–8]. It has been stated that BMPs are able to stimulate local undifferentiated mesenchymal cells to transform into osteoblasts (osteoinduction), and lead to early bone formation [9–12]. More study is still necessary to identify which BMPs have higher osteoinductive properties and are more efficient in clinical application. Based on the recent literature, it seems that bone tissue engineering is the newest option for promoting and accelerating the healing potential of bone defects [13]. In bone tissue engineering, it is possible to combine synthetic scaffolds with biological biomaterials to stimulate cell infiltration and new bone formation and to enhance the healing process. In this regard, gene therapy (transfer of genes that code growth factors such as BMPs to target cells with the help of a plasmid or viral vector) may provide promising results, although concerns over trans-infection of the target cells with the gene are an unresolved issue [14–17].

Stem cells such as adipose-derived stem cells (ASCs) can differentiate into the osteogenic lineage. Furthermore, osteoid matrix formation has been observed when osteo-induced human ASCs were seeded onto hydroxyapatite/tricalcium phosphate scaffolds and implanted subcutaneously in nude mice [18]. Cowan et al. [19] demonstrated that osteo-induced ASCs along with the apatite-coated polylactic-coglycolic acid scaffold could repair a critical-sized calvarial defect of a mouse model. Meanwhile, Dudas et al. [20] showed that ASCs in combination with gelatin gel could repair a non-critical-sized defect in a rabbit model with a follow-up of 6 weeks. All these results indicate that ASCs could be an alternative cell source for bone engineering [21].

Hydroxyapatite, a crystalline phase of calcium phosphate found naturally in bone minerals, has shown tremendous promise as a graft material. It exhibits initial mechanical rigidity and structure, and demonstrates osteoconductive as well as angiogenic properties in vivo [22]. Additionally, fabricated porous hydroxyapatite scaffolds have been reported to promote a strong mechanical interlock with the host bone tissue [22, 23]. Since the extent of bony ingrowth within the scaffold, the functionality of newly regenerated bone tissue, and the development of a vascularized network within the scaffold are dictated by the porous scaffold architecture, extensive studies have been performed to optimize new biomaterials needed for maximal bone tissue integration [24, 25].

The presence of transforming growth factor β (TGF-β) in growth plate [26] and BMPs 2 and 7 in human and rat fetal growth plate have been identified previously [27]. These proteins promote the chondroblastic differentiation of mesenchymal cells, followed by new bone synthesis by endochondral osteogenesis [28, 29]. A previous study proved that segmental bovine growth plate grafting has potential osteoinductive properties [30]. More recently, another study showed ectopic osteoinductive properties of calf fetal growth plate in a rat sub-muscular model [31] and bone healing enhancement in a rabbit bone defect model [32]. The present study was designed to evaluate the bone healing properties of demineralized calf fetal growth plate concurrent with hydroxyapatite in a critical-sized bone defect experimental rabbit model.

## Materials and methods

Fifteen New Zealand white rabbits (12 months old, mixed sex, weighing 2.0 ± 0.5 kg) were kept in separate cages, fed a standard diet and allowed to move freely during the study. The animals were randomly divided into three equal groups: DCFGP group (*n* = 5), hydroxyapatite–DCFGP group (*n* = 5) and hydroxyapatite group (*n* = 5 group). All the animals were anesthetized by intramuscular administration of 40 mg/kg ketamine hydrochloride and 5 mg/kg xylazine. The right forelimb of all animals was prepared aseptically for operation. A 5-cm incision was made craniomedially in the skin of the forelimb and the radius was exposed by dissecting the surrounding muscles. An osteoperiosteal segmental defect was then created on the middle portion of each radius at least twice as long as the diameter of the diaphysis, for creation of a nonunion model [33]. As the diameter of the radius of the adult New Zealand albino rabbit is about 5–6 mm, the radial defect was 10–12 mm long. Therefore, an approximately 10-mm segmental defect was created in the middle portion of each radius as a critical-sized bone defect. The defect in the animals in the hydroxyapatite group was filled with 1 mg of hydroxyapatite segments (OS Satura^®^, Isotis Co, Netherlands). In the hydroxyapatite–DCFGP group the bone defect was filled with 0.5 mg of hydroxyapatite segments and 0.5 mg of DCFGP powder, while the defects in the animals in the DCFGP group were filled with 1 mg of DCFGP powder. The animals were housed in compliance with our institution’s guiding principles “on the care and use of animals”. The local Ethics Committee for animal experiments approved the design of the experiment.

A 6-month old bovine fetus was collected from the local slaughter house. Metacarpal bones were dissected aseptically from the fetal calf (Holstein) and all soft tissues were removed. Radiographs were taken to determine the growth plate’s margins and limitations. With an oscillating osteotome, proximal and distal growth plates were cut and retrieved under aseptic conditions. The retrieved growth plate was then sliced. The demineralization process was performed as described by Reddi and Huggins [34]. The harvested growth plates were cleaned of soft tissue and marrow, washed in sterile distilled water with continuous stirring, then washed three times in 95 % ethanol for 15 min, rinsed in ether for 15 min, and finally air-dried overnight. The cleaned and dried growth plates were milled (Universal Mill A-20; Tekmer Co, Cincinnati, OH, USA) to obtain 400–700-μm granules and then demineralized in 0.6-N HCl three times for 1 h (50 ml HCl per g of bone). The growth plate powder was rinsed with several changes of sterile distilled water to adjust the pH, three times in 95 % ethanol and once in ether. The growth plate powder was air-dried and stored in sterile plastic containers at 4 °C until being used for implantation. This entire process was performed under sterile conditions (except for the milling) and a sample was cultured to demonstrate that specimens contained no bacterial or fungal contamination.

To evaluate bone formation, union and remodeling of the defect, radiographs of each forelimb were taken postoperatively at the 2nd, 4th, 6th and 8th weeks post-injury. The results were scored using the modified Lane and Sandhu scoring system [35] (Table 1).

**Table 1:** Modified Lane and Sandhu radiological scoring system

Bone formation	
No evidence of bone formation	0
Bone formation occupying 25 % of the defect	1
Bone formation occupying 50 % of the defect	2
Bone formation occupying 75 % of the defect	3
Bone formation occupying 100 % of the defect	4
Union (proximal and distal evaluated separately)	
No union	0
Possible union	1
Radiographic union	2
Remodeling	
No evidence of remodeling	0
Remodeling of medullary canal	1
Full remodeling of cortex	2
Total points possible per category	
Bone formation	4
Proximal union	2
Distal union	2
Remodeling	2
Maximum score	10

Animals were killed, and radius bones were explanted on the 56th postoperative day for gross and histopathological signs of healing. In gross evaluation, examination and blinded scoring of the specimens included presence of bridging bone, indicating a complete union (+3 score), presence of cartilage, soft tissue or cracks within the defect indicating a possible unstable union (+1 or +2 score), or complete instability at the defect site indicating no union (0 score).

For histopathological evaluation, sagital sections containing the defect were cut with a slow-speed saw from the harvested and dissected bones. Each slice was then fixed in 10 % neutral buffered formalin. The formalin-fixed bone samples were decalcified in 15 % buffered formic acid solution and processed for routine histological examination. Two sections 5 µm in thickness were cut from the centers of each specimen and were stained with hematoxylin and eosin. The sections were blindly evaluated and scored by two pathologists according to Emery’s scoring system [36] and based on this scoring system the defects were evaluated as follows: if the gap was empty (score = 0), if the gap was filled with fibrous connective tissue only (score = 1), with more fibrous tissue than fibrocartilage (score = 2), more fibrocartilage than fibrous tissue (score = 3), fibrocartilage only (score = 4), more fibrocartilage than bone (score = 5), more bone than fibrocartilage (score = 6) and filled only with bone (score = 7).

## Results

There was no intra-operative or postoperative death during the study. None of the rabbits sustained an ulna bone fracture at the radius bone defect.

**Fig. 1:**
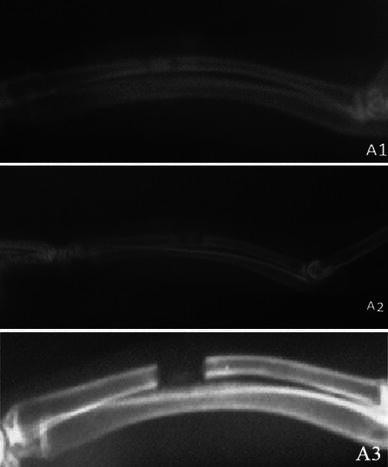
Radiographs at 2nd week: **A1** hydroxyapatite group, **A2** hydroxyapatite–DCFGP group and **A3** DCFGP group

**Fig. 2:**
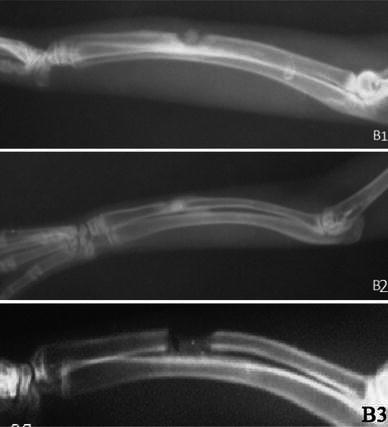
Radiographs at 4th week, **B1** hydroxyapatite group, **B2** hydroxyapatite–DCFGP group and **B3** DCFGP group

**Fig. 3:**
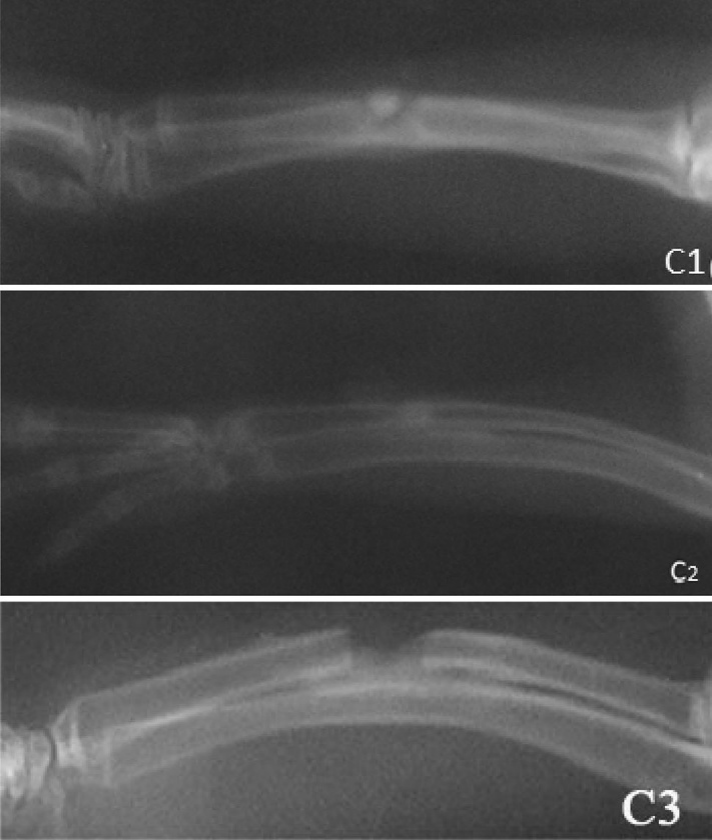
Radiographs at 6th week, **C1** hydroxyapatite group, **C2** hydroxyapatite–DCFGP group and **C3** DCFGP group

**Fig. 4:**
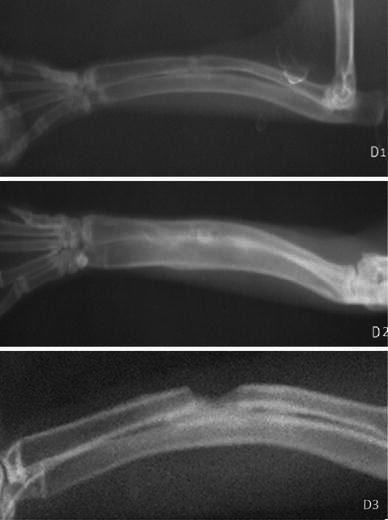
Radiographs at 8th week, **D1** hydroxyapatite group, **D2** hydroxyapatite–DCFGP group and **D3** DCFGP group

At the 2nd, 4th and 6th postoperative weeks, the radiographs did not show any significant differences between any of the groups, whereas at the 8th postoperative week the radiographs showed significant differences between the groups (*p* < 0.05) (Figs. 1, 2, 3, 4; Table 2). The hydroxyapatite–DCFGP group was significantly (*p* < 0.05) superior to the hydroxyapatite and DCFGP groups. There were no significant differences between the DCFGP and hydroxyapatite groups on any postoperative day (Figs. 1, 2, 3, 4; Table 2).

**Table 2:** Radiographical findings for healing of the bone defect (sum of the radiological scores) at various post-operative intervals

Postoperative weeks	Median (min–max)			*P* ^a^
	Hydroxyapatite group (*n* = 5)	Hydroxyapatite–DCFGP group (*n* = 5)	DCFGP group (*n* = 5)	
2	1 (0–3)	3 (1–4)	1 (0–3)	0.08
4	4 (3–8)	7 (4–10)	5 (3–6)	0.1
6	5 (2–8)	8 (6–10)	5 (4–7)	0.07
8	6 (4–9)	9 (8–10)^b^	5 (4–7)	**0.01**

Significant *P* values are shown in *bold face*

^a^Kruskal–Wallis non-parametric analysis of variance

^b^Compared with hydroxyapatite group (*p* = 0.02) and DCFGP group (*p* = 0.008) by Mann–Whitney *U* test. Hydroxyapatite–DCFGP group was significantly (*p* < 0.05) superior to hydroxyapatite and DCFGP groups

The defect areas of the rabbits in all groups showed various amounts of new bone formation; the union scores of the rabbits in the hydroxyapatite–DCFGP group were not statistically superior to those of the animals in the hydroxyapatite and DCFGP groups (Table 3).

**Table 3:** Bone measurements at macroscopic and microscopic level

Bone type evaluation	Median (min–max)			*P* ^a^
	Hydroxyapatite group (*n* = 5)	Hydroxyapatite–DCFGP group (*n* = 5)	DCFGP group (*n* = 5)	
Macroscopic union^a^	3 (2–2)	3 (2–3)	2 (2–2)	0.1
Microscopic evaluation^b^	6 (4–7)	7 (5–7)	5 (4–7)	0.1

^a^Complete union (+3 score), presence of cartilage, soft tissue or cracks within the defect indicating a possible unstable union (+ 1 or +2 score), complete instability at the defect site indicating nonunion (0 score)

^b^Empty (0 score), fibrous tissue only (1 score), more fibrous tissue than fibrocartilage (2 score), more fibrocartilage than fibrous tissue (3 score), fibrocartilage only (4 score), more fibrocartilage than bone (5 score), more bone than fibrocartilage (6 score) and bone only (7 score)

At the histopathological level, the defects in the animals in the hydroxyapatite–DCFGP and hydroxyapatite and DCFGP groups did not show significant differences on statistical analysis (*p* > 0.05) (Table 3).

**Fig. 5:**
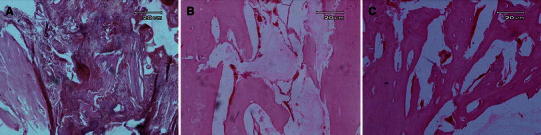
Micrographs of the injured bones after 8 weeks. Regenerated bone with typical structure of trabecular bone is seen in the defect in the hydroxyapatite group (**a**, ×10), hydroxyapatite–DCFGP group (**b**, ×10) and DCFGP group (**c**, ×10) (hematoxylin and eosin staining)

The defects in all rabbits in the three groups were filled with mature cortical bone (Fig. 5). Normal trabecular and woven bone were uniformly formed within the defects and the regenerated bone completely spanned the defect and mostly produced full histological union (Fig. 5). No significant inflammatory response was evident in the lesions in the animals of different groups at 8 weeks post injury, although it may have been present earlier.

## Discussion

To evaluate the bone healing potential of a combination of hydroxyapatite and DCFGP, a defect model was established in the radius bone of rabbits. This model has previously been reported suitable because there is no need for internal or external fixation which influences the healing process [37]. Chaubey et al., in a murine model, showed that bone defect healing occurred at 2 weeks and were completely healed by 5 weeks, with biomechanical properties not significantly different from normal controls. However, critical-sized defects showed no healing by histology or micro-computed tomography. These nonunion fractures also displayed no torsional stiffness or strength in 10 out of 12 cases [38]. In the present study the segmental defect was created in the middle portion of the radius, as long as 10 mm, for inducing a nonunion defect and to prevent spontaneous and rapid healing [33]. The hypothesis was on the basis that the addition of DCFGP to a mixture of particulate hydroxyapatite in a critical-sized defect in the radius bone of rabbit could have a positive effect on bone formation.

The results of the radiological examinations showed that bone healing was enhanced when DCFGP was used concurrently with hydroxapatite. Recently, a study indicated that satisfactory ectopic bone formation occurred in a submuscular rat model with xenogenic demineralized bovine fetal growth plate, and complications were not identified [31]. In addition, in two previous studies segmental calf fetal growth plate was grafted in the radial bone defect and a positive bone healing process was observed by investigators [30, 39]. A more recent study showed favorable bone defect healing with DCFGP in a rabbit model [32]. In our study too, the DCFGP group showed good bone healing the same as other groups on histopathological evaluation. The presence of TGF-β in the growth plate [26] and BMPs 2 and 7 in human and rat fetal growth plate [27] has been identified. These proteins promote the chondroblastic differentiation of mesenchymal cells, followed by new bone synthesis by endochondral osteogenesis [29]. The primary osteoinductive component of demineralized bone matrix (DBM) is a series of low-molecular-weight glycoproteins that includes the BMPs. The decalcification of cortical bone exposes these osteoinductive growth factors buried within the mineralized matrix, thereby enhancing the bone formation process. These proteins promote the chondroblastic differentiation of mesenchymal cells, followed by new bone synthesis by endochondral osteogenesis [29, 40]. We propose that in our study calf fetal growth plate demineralization led to the exposure of TGF-β and BMPs 2 and 7 in the injured site, and therefore the bone healing process in the DCFGP group was superior to the control group. In the present study DCFGP did not elicit any inflammatory reaction in the grafted site in the DCFGP and hydroxyapatite–DCFGP groups. It has been reported that the demineralization process destroys the antigenic materials in bone, so that the DBM becomes less immunogenic and does not induce an immunological reaction by the host [41]; we did not observe any inflammatory reaction throughout the histopathological evaluation.

Hydroxyapatite, a crystalline phase of calcium phosphate found naturally in bone minerals, has shown tremendous promise as a graft material. It exhibits initial mechanical rigidity and structure, and demonstrates osteoconductive as well as angiogenic properties in vivo [22, 42, 43]. In osteoperiosteal gaps bridged with hydroxyapatite only, the porosities were invaded with fibrous tissue or fibrocartilage tissues more than bone tissues. Occasionally, bone formation was observed in direct contact with hydroxyapatite, confirming its osteoconductive ability, but it was insufficient to allow union. These findings are similar to those reported using hydroxyapatite [25]. When the gap reaches a critical size, the osteoconductive properties of the material are insufficient to fill the gap with formation of new bone [44]. This model therefore proved to be adequate for evaluating hydroxyapatite as a scaffold for DCFGP. More unexpected is the formation of the cortex and medullary canal together with mature lamellar bone observed in most of the cases. The previous in vitro studies have shown that artificial bone graft materials support the attachment, growth and differentiation of the bone-marrow stromal cells [45].

The rate of BMP release relies on its molecular weight, its conformation and its solubility [46]. Gene therapy-based strategies have also been introduced to improve BMP delivery and their effectiveness at the target site [47]. This technology provides the gene for the protein and results in a higher and more constant level of BMPs for a sustained time period [48]. To include the gene for BMPs into the target cell, a delivery vehicle or vector is needed, viral or non-viral [47].

Sohier et al. [49] investigated the efficiency of BMP-2 delivered by macroporous beta-tricalcium phosphate (β-TCP) scaffolds. The scaffolds, loaded with 15 and 30 µg of BMP-2, were implanted into the femoral defects and the back muscles of rabbits, respectively. Bone was formed within the BMP-2-loaded scaffold pores, both in the back muscles and bone defects, independent of the implant site effect. The results of that study indicated the efficacy and suitability of β-TCP scaffolds as BMP-2 carriers for bone regeneration. In another study, the in vitro and in vivo effectiveness of an absorbable collagen sponge (ACS) with 72 µg rhBMP-2 (BMPC) and fibrin matrix with 10 µg rhBMP-2 (BMPF) were compared with the ACS alone, fibrin alone, and empty groups. BMP-2 release was significantly higher in the BMPF group than in the BMPC group. The bone union of femoral defects and the bone volume were higher in the BMPC and BMPF groups than in other groups. Interestingly, fibrin matrix even with a seven-fold lower concentration of BMP-2 provided equivalent results to collagen sponge. According to those results, it seems that fibrin matrix could be an excellent carrier for BMP-2 [50].

Jun et al. [51] fabricated a silica xerogel–chitosan hybrid for incorporating BMP-2 onto a porous hydroxyapatite (HA) scaffold. They evaluated the biological properties of the hybrid coating incorporated with BMP-2, in terms of the release behavior of BMP-2, and also its in vivo performance on calvarial defects in rabbits. The BMP-2-loaded hybrids significantly enhanced new bone formation in comparison to the pure porous HA scaffolds without BMP-2. Indeed, the incorporation of BMP-2 into the hybrid promoted the osteoinductive properties of the HA scaffold. They introduced the silica xerogel–chitosan hybrid as a promising candidate for improving osteogenic properties of the HA scaffold with the constant and prolonged release of BMP-2.

The findings of the present study suggest that hydroxyapatite is a suitable resorbable carrier for DCFGP in vivo. It serves as a substrate to promote attachment and growth of the stem cells of the bone marrow, and as a template to guide bone morphogenesis in a clinically relevant volume. According to this phenomenon, in our study the hydroxyapatite–DCFGP group showed good enhancement of bone healing in comparison with hydroxyapatite and DCGP on radiological evaluation at the 8th postoperative week. Alper and colleagues [52] designed a study to identify the role of HA and DBM combination in fracture healing. After creating 5-mm segmental defects in the radii of the rats, defects were grafted using DBM, HA and DBM/HA mixture. After eight weeks of fracture healing, the radii were investigated histologically, and the HA group was found to have worse results when compared to the control group. They stated that DBM alone was an osteogenetic material for healing in non-union models of the rats, but that using HA in conjunction caused these effects to fade away. Moore and colleagues grafted wide ulnar defects of dogs with HA/TCP mixtures. They used a half-and-half mixture of autogenous cancellous bone and HA/TCP mixtures for one of the other groups, and pure autogenous cancellous bone for the control group. After six months, all groups were evaluated radiologically, mechanically and histhologically, and it was found that six dogs which were only grafted with the HA/TCP mixture showed fibrous nonunion, whereas other groups displayed union. As a result, they suggested that HA/TCP should be used as a mixture with autogenous grafts [53].

Hopp and colleagues [54] found similar results. They designed an experiment in which the ulnar defects of rats were grafted by HA, DBM, HA/DBM, autogenous bone graft and allogenic bone graft in five different groups. At the 6th week, they found that the plain HA group scored worse than the control group, whereas all other groups displayed better scores.

In our study, macroscopic and histopathological evaluation did not reveal any significant differences between the three groups after 8 weeks and showed favorable bone healing scores in the three groups. The authors proposed that there might be some differences during the earlier stages of the healing but by 8 weeks post-injury the three groups had reached almost the same level. As a result, similar macroscopic and microscopic healing responses were observed and further studies are needed before considering DCFGP-HA as an alternative to HA alone in clinical practice, especially considering the potential biological risks of animal-derived materials.
